# A YOLO11-MSC-Based Method for Small Pest Detection in Insect Pest Monitoring Lamp Images

**DOI:** 10.3390/s26144576

**Published:** 2026-07-19

**Authors:** Zhiyong Li, Zuxiang Lin, Xulin Liu, Yuzhu Li

**Affiliations:** 1 College of Information Engineering, Sichuan Agricultural University, Yaan 625015, China; liuxulin@stu.sicau.edu.cn (X.L.);; 2 College of Mechanical and Electrical Engineering, Sichuan Agricultural University, Yaan 625015, China; 2025117004@stu.sicau.edu.cn

**Keywords:** agricultural pest detection, deep learning, YOLO11, insect pest monitoring lamp, small object detection

## Abstract

**Highlights:**

**What are the main findings?**

- A lightweight detection framework, YOLO11-MSC, was developed to improve small-pest detection in high-resolution insect pest monitoring lamp images.
- The proposed OCAS strategy, together with the MNS and C2iBRA modules, enhances small-object representation, multi-scale feature extraction, and suppression of complex background interference.
- The SGAS inference strategy effectively bridges slice-based model training and full-image inference on original high-resolution images.

**What are the implications of the main findings?**

- The proposed method provides a robust and computationally efficient solution for detecting small and densely distributed pests in complex monitoring scenarios.
- The framework supports accurate pest identification and quantity estimation, thereby offering practical technical support for intelligent pest monitoring and precision pest management.

**Abstract:**

To address the challenges of small pest target sizes, dense distributions, strong background interference, and missed detection of small objects in insect pest monitoring lamp images, this study proposes YOLO11-MSC, a lightweight agricultural pest detection model based on an improved YOLO11. First, an Object-Centric Adaptive Slicing (OCAS) strategy is designed to construct the Insect35_OCAS dataset, which increases the relative proportion of small pest targets in the input images while reducing redundant background interference. Second, the Multi-path Nonlinear Star Aggregation (MNS) module and the C2-based Improved Bi-Level Routing Attention (C2iBRA) module are introduced to enhance fine-grained feature extraction for multi-scale pest targets and improve background suppression in complex scenes. Finally, a Semantic-Guided Adaptive Slicing (SGAS) inference strategy is developed to transfer the sliced-image training model to full-image inference on original high-resolution insect pest monitoring lamp images. Experimental results show that YOLO11-MSC achieves an mAP@0.5, Precision, and Recall of 95.2%, 91.8%, and 93.0% on the Insect35_OCAS dataset, respectively, improving the baseline YOLO11n by 1.3, 1.0, and 1.2 percentage points. The model contains only 3.4 M parameters and requires 7.8 Giga Floating-Point Operations (GFLOPs). When combined with SGAS, YOLO11-MSC achieves an mAP@0.5, Precision, and Recall of 86.4%, 81.2%, and 90.1% on the original full-image dataset, respectively. These results demonstrate that the proposed method effectively improves the detection accuracy of small pests and the full-image inference capability in complex insect pest monitoring lamp scenarios while maintaining low model complexity, providing technical support for intelligent agricultural pest monitoring and precision control.

## 1. Introduction

Plant diseases and insect pests are among the major threats to global food security. According to projections for 2026, 23 major pests and diseases affecting staple crops, rapeseed, soybean, and vegetable crops in China are expected to maintain a relatively high incidence level, covering an area of approximately 150.011 million hectares nationwide [1]. In the face of severe yield-loss risks, timely and accurate detection and identification of crop diseases and insect pests are of great importance. As a key component of agricultural pest monitoring, the accuracy and timeliness of pest forecasting directly affect the implementation of subsequent control measures. However, traditional pest monitoring relies heavily on manual inspection, which is labor-intensive, inefficient, and vulnerable to background interference, making it difficult to meet the requirements of real-time and accurate pest forecasting [2].

In recent years, automated trapping devices, machine vision, and deep learning technologies have made significant progress in the automatic identification of pests captured by insect pest monitoring lamps [3,4,5]. Lima et al. [6] systematically reviewed automatic pest monitoring technologies based on sensors, image recognition, machine learning, and the Internet of Things, and indicated that automatic trapping and visual recognition systems have great potential for early pest monitoring and precision pest control. Yao et al. [7] developed an automatic monitoring system for rice light-trap pests, which enabled image acquisition, uploading, recognition, and counting of light-trapped pests. A one-year field trial demonstrated a high correlation between automatic recognition results and manual counting results. Sun et al. [8] designed an intelligent monitoring system for migratory pests based on searchlight trapping and machine vision. Through searchlight trapping, conveyor-belt dispersion, image acquisition, and cloud-based recognition, the system achieved automatic identification and counting of migratory pests such as the rice leaf folder, white-backed planthopper, and brown planthopper. Bjerge et al. [9] proposed an automated light-trap monitoring device for Lepidoptera insects, using cameras and deep learning methods to track, count, and identify nocturnally attracted insects, thereby providing an effective solution for automatic insect monitoring in light-trap scenarios.

In terms of detection algorithms, extensive studies have been conducted to address fine-grained classification, multi-class detection [10], small-object recognition [11], and background interference suppression [12]. Early object detection methods mainly relied on handcrafted features and traditional machine learning models, which made them difficult to adapt to complex insect pest monitoring images characterized by small target sizes, strong background interference, and high inter-class similarity. With the development of deep learning, two-stage detectors such as Faster R-CNN have improved detection accuracy through region proposal generation and refined classification. However, their large number of parameters and high computational cost make them less suitable for edge deployment in insect pest monitoring lamp scenarios. In contrast, YOLO-based one-stage detectors offer advantages in detection speed and model complexity and have therefore been widely applied to agricultural pest detection tasks. Yao et al. [13] proposed a fine-grained recognition model for agricultural light-trapped pests based on a bilinear attention network, achieving automatic identification of 19 visually similar light-trapped pest species with an average recognition accuracy of 94.9%. Lv et al. [14] proposed an improved YOLOv3 detection method combined with image augmentation and instance augmentation to address class imbalance, object occlusion, and inter-class similarity in light-trap pest images, effectively improving the detection performance for corn pests in light-trap images. Zhang et al. [15] proposed a lightweight detection model, AgriPest-YOLO, which improved light-trapped pest detection by integrating coordinated local attention, a grouped spatial pyramid pooling module, and Soft-NMS. The model achieved an mAP of 71.3% on a dataset containing approximately 25,000 images from 24 pest categories. Wang et al. [16] proposed ASP-Det for appearance-similar light-trapped pest detection and constructed the PestNet-AS dataset. By defining appearance-similar pest detection from the perspectives of texture similarity and scale similarity, their study demonstrated that light-trap pest images involve not only small-object detection challenges but also difficulties in fine-grained category discrimination. Wen et al. [17] proposed Pest-YOLO to address large-scale, multi-class, dense, and tiny pest detection and counting, further indicating that pest detection tasks commonly suffer from large scale variations, dense object distributions, and class imbalance.

Although previous studies have advanced automatic pest recognition in insect pest monitoring lamp scenarios and constructed agricultural pest image datasets such as AgriPest and Pest24, which provide important data support for multi-class pest recognition, detection, and model performance evaluation, as well as useful benchmarks for comparing different detection models under complex backgrounds, inter-class similarity, and scale variation, several challenges still remain in practical monitoring lamp images. First, original pest monitoring images usually have high resolution, whereas individual pest targets occupy only a small proportion of the whole image. Directly resizing the original image before feeding it into a detection model can easily lead to the loss of texture, edge, and geometric structure information. Second, light-trap images are often affected by overlapping insect bodies, incomplete limbs, background stains, uneven illumination, and visually similar categories, which may result in missed detections and false detections. In addition, most existing methods focus on sliced images or local image detection, while insufficient attention has been paid to how a slice-trained model can be effectively transferred to original high-resolution full-image inference scenarios. Therefore, small-object pest detection in insect pest monitoring lamp scenarios still requires further optimization in terms of dataset construction, feature extraction, attention modeling, and full-image inference strategies.

To address the above challenges, this study proposes an agricultural pest detection method for insect pest monitoring lamp scenarios. The main contributions of this study are summarized as follows:

(1) A padding-free object-centered adaptive slicing strategy, termed OCAS, is designed to alleviate the small-object scale problem and construct a high-quality pest detection dataset, Insect35_OCAS, thereby improving the relative scale and feature representation of small pest targets.
(2) To overcome the limitations of the C3K2 module in YOLO11, such as its relatively simple feature extraction path and insufficient representation capability for multi-scale pest targets, an Multi-path Nonlinear Star Aggregation (MNS) module integrating multi-path heterogeneous aggregation and high-order nonlinear interaction is proposed. This module enhances the model’s feature extraction capability for small-scale, overlapping, and morphologically complex pest targets.
(3) A C2-based Improved Bi-Level Routing Attention (C2iBRA) module is introduced by incorporating the bi-level routing attention mechanism into the C2PSA-like structure. To make it more suitable for pest monitoring lamp images, scale-aware preprocessing is combined with BRA to improve target-focused attention and suppress background interference caused by stains, uneven illumination, and visually similar non-target regions.
(4) A semantic-guided adaptive slicing inference strategy, termed SGAS, is designed to effectively transfer the slice-trained model to original high-resolution pest monitoring images.

## 2. Materials and Methods

### 2.1. Pest Image Dataset Construction

#### 2.1.1. Data Acquisition

**Figure 1:**
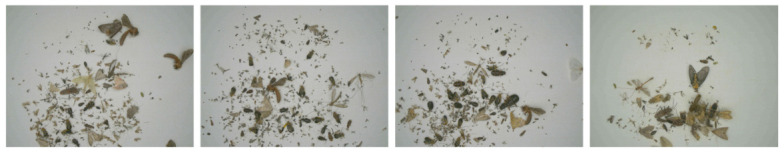
Representative images from the dataset.

The data used in this study were collected using an intelligent insect pest monitoring lamp system independently developed by Chengdu Biang Technology Co., Ltd. (Chengdu, China). The system was continuously deployed for seven years in major agricultural production regions of China, including Sichuan, Yunnan, Xinjiang, and Shaanxi, accumulating a large number of field pest monitoring images. The monitoring lamps attract insects to the collection plate using light sources with specific wavelengths, and high-resolution cameras are used to automatically capture images at regular intervals. To ensure the geographical and seasonal representativeness of the dataset, samples were balanced across different regions and months from the original image data. All collected images were saved in JPG format with a resolution of 4000 × 3060 pixels. Representative images collected by the system are shown in Figure 1.

#### 2.1.2. Dataset Construction

(1) Image annotation and category composition

All images were annotated for object detection using LabelImg (https://github.com/HumanSignal/labelImg (accessed on 16 July 2026)) [18], and the category labels were named using the Chinese pinyin of the corresponding pest species. According to the annotation protocol, each bounding box was required to tightly enclose the outer contour of the pest while retaining a margin of approximately 2–3 pixels. For samples with clearly visible insect body structures, detached or incomplete limb regions were additionally annotated to enhance the model’s ability to recognize pests with varying morphologies and partial occlusions. To ensure annotation quality, a three-level review mechanism was adopted. First, junior annotators completed the initial annotation. Then, domain experts randomly inspected, verified, and corrected the annotated categories and bounding boxes. Finally, random sampling checks were conducted to ensure annotation consistency.

**Figure 2:**
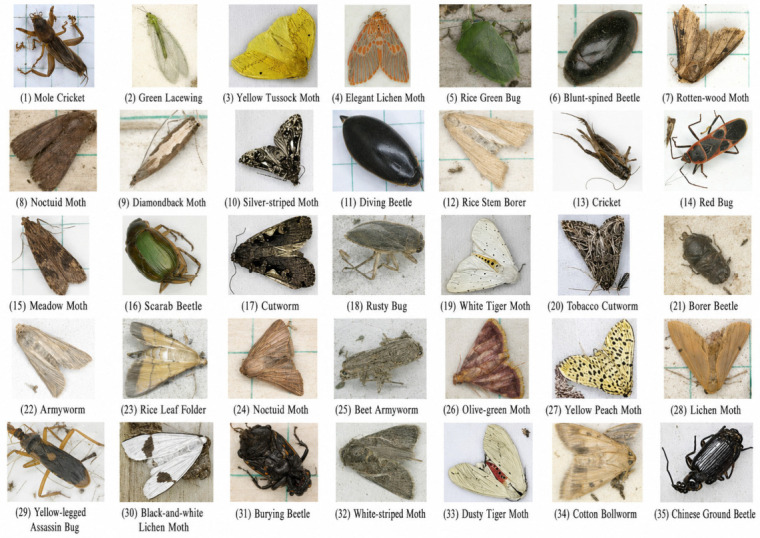
Representative examples of different pest categories in the dataset.

Under the guidance of agricultural experts, 35 key pest species with significant impacts on crop yield were selected, and a dedicated agricultural pest detection dataset was constructed through accurate annotation. The dataset covers 35 pest categories belonging to multiple insect orders, including Neuroptera, such as green lacewing, and Coleoptera, such as scarab beetle, burying beetle, Chinese ground beetle, blunt-spined beetle, diving beetle, and borer beetle. The complete pest categories are shown in Figure 2.

(2) Slicing parameter selection and construction of Insect35_OCAS

The original insect pest monitoring lamp images have high spatial resolution, whereas most pest targets occupy only a small proportion of the entire image. Directly resizing the original images and feeding them into the detection model may lead to the loss of texture, edge, and morphological information of small-scale pests, thereby increasing the risk of missed detections and false detections. Therefore, an image slicing strategy was adopted to increase the relative scale of pest targets in the input images and provide more effective feature representations for subsequent small object detection.

**Figure 3:**
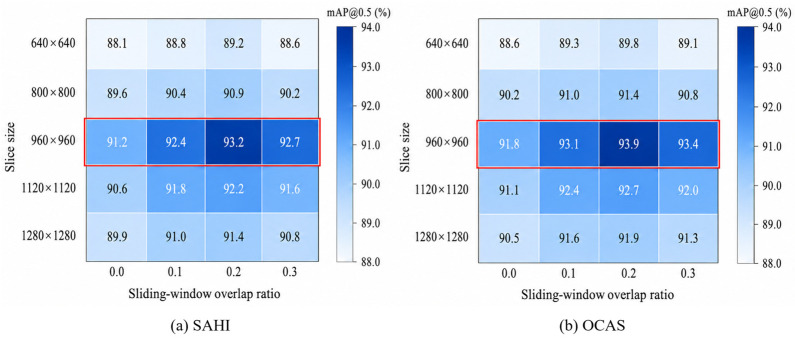
Detection performance comparison under different slice sizes and sliding-window overlap ratios. The red boxes indicate the selected slice size of 960 × 960 pixels, which achieved the best overall detection performance.

To determine the optimal window size for the subsequent slicing strategy, a parameter analysis was conducted using both the fixed sliding-window slicing strategy based on Slicing Aided Hyper Inference (SAHI) [19] and the proposed Object-Centric Adaptive Slicing (OCAS) strategy under different combinations of slice sizes and overlap ratios. The results are shown in Figure 3. Both SAHI and OCAS achieved their best detection performance when the slice size was set to 960 × 960 and the overlap ratio was 0.2. Under this setting, SAHI obtained an mAP@0.5 of 93.2%, whereas OCAS achieved 93.9%, representing an improvement of 0.7 percentage points. Although the improvement was relatively modest, OCAS showed more stable performance under different slicing parameters, indicating that the object-centered slicing strategy can better preserve pest target integrity and reduce interference from redundant background regions. Considering detection accuracy, target integrity, and computational cost, 960 × 960 was ultimately selected as the standard slice size for the OCAS strategy. It should be noted that the fixed sliding-window strategy was mainly used to determine the optimal slice scale and overlap parameter, whereas OCAS further reduces irrelevant background regions through target-centered localization and increases the effective pixel proportion of pest targets in the input images.

On this basis, an object-centered non-padding adaptive slicing strategy, namely Object-Centric Adaptive Slicing (OCAS), was adopted to construct the Insect35_OCAS dataset. The detailed procedure is as follows. First, the original insect pest monitoring lamp images and their corresponding annotation files were read to obtain the bounding box information of each pest target. Then, the center point of each target was calculated based on its bounding box, and this point was used as the slicing center to crop a local region from the original image using a 960 × 960 pixel window. When the cropping region exceeded the boundary of the original image, OCAS did not use fixed-value padding; instead, only the valid region within the original image was retained to avoid introducing additional interference caused by artificially padded boundaries. Finally, the bounding box coordinates in the sliced images were transformed accordingly, invalid annotations outside the sliced region were removed, and valid targets with their corresponding category labels were retained.

Compared with the fixed sliding-window strategy, OCAS can generate training samples more precisely around pest targets. While preserving target integrity, it increases the pixel proportion of small-scale pests in the input images and reduces the influence of complex background regions during model learning. Based on the OCAS strategy, the Insect35_OCAS dataset containing 69,625 sliced images was constructed, and the detailed sample information is presented in Table A1. This dataset effectively alleviates the weakening of small-object features in high-resolution insect pest monitoring lamp images, enabling the model to focus more on pest body regions and improving its detection capability for small-scale, densely distributed, and morphologically complex pest targets.

Specifically, the original images were first divided into training, validation, and test sets at a ratio of 7:2:1. OCAS was then independently applied to each subset to generate the corresponding training, validation, and test slices. In this way, all slices derived from the same original image were kept within the same subset, thereby avoiding potential data leakage. The training set was used for model parameter optimization; the validation set was used for performance evaluation and model selection during training, and the test set was used for final detection performance evaluation.

### 2.2. Overall Architecture of YOLO11-MSC

YOLO11 [20] is a single-stage object detection model released by the Ultralytics team in 2024. Built upon YOLOv8 [21], it incorporates Transformer-based attention mechanisms, dynamic label assignment strategies, and refined model scaling methods, thereby improving detection accuracy while maintaining high inference speed. Its overall architecture follows the typical three-stage design consisting of a backbone, a neck, and a detection head. Specifically, lightweight self-attention modules, adaptive multi-scale feature fusion mechanisms, and a decoupled detection head are introduced to enhance the model’s feature extraction and localization capabilities for multi-scale targets.

**Figure 4:**
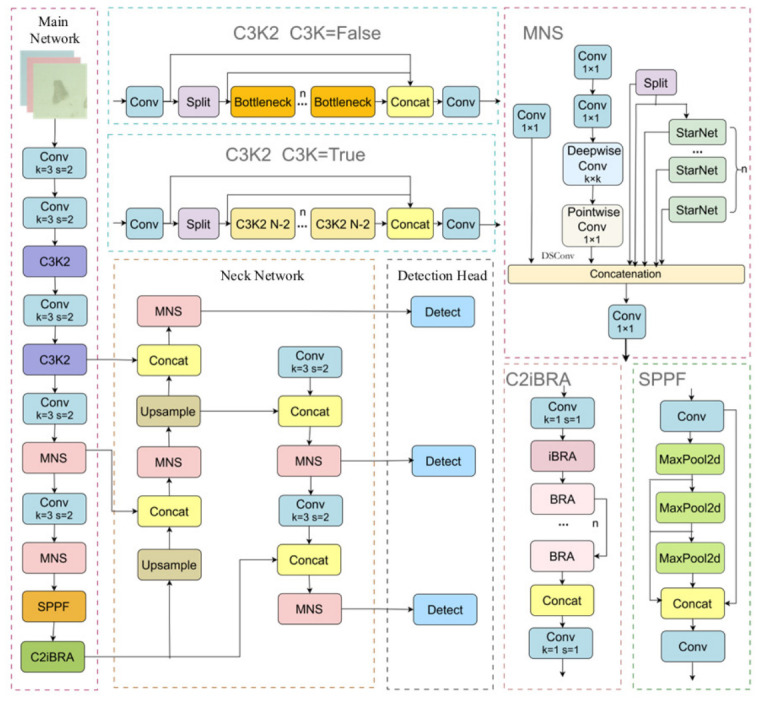
Structure of the YOLO11-MSC algorithm.

However, when applied to agricultural pest detection, YOLO11 still faces several challenges, including high inter-class similarity, insufficient feature representation for small targets, and relatively high model redundancy. To address these issues, an improved model, named YOLO11-MSC, is proposed in this study, and its architecture is shown in Figure 4. Specifically, in the deep layers of the backbone network, the original C3K2 module is replaced with the proposed MNS module. The MNS module inherits the multi-path parallel structure of MANet to improve feature diversity, and further embeds the Star module, which replaces conventional linear fusion operations with element-wise multiplication. This design enables high-dimensional nonlinear feature representation while maintaining low computational overhead. In the neck network, the C2PSA module is replaced with the proposed C2iBRA module, which optimizes attention allocation and enhances the model’s ability to distinguish pest foreground regions from complex backgrounds. Experimental results demonstrate that YOLO11-MSC effectively suppresses inter-class false detections and background interference while significantly reducing the number of model parameters. Moreover, the attention response regions more accurately cover the pest bodies, leading to notable improvements in both missed detection and false detection rates compared with the baseline model.

### 2.3. Improvements to the YOLO Algorithm

#### 2.3.1. MNS Module

Mixed Aggregation Network (MANet) [22], as a core feature extraction module in the backbone network of Hyper-YOLO, enhances feature diversity and representation capability by integrating multiple convolutional structures. However, its core CN module essentially relies on a combination of linear convolution and activation functions, and its feature transformation capability is constrained by limited nonlinear interactions. This limitation makes it difficult to effectively distinguish pest categories with similar morphologies. To address this issue, a mixed aggregation network module, termed MNS, is proposed in this study. Based on the multi-path parallel structure of MANet, MNS replaces the CN module in one branch with a Star module [23]. The Star module performs interaction between the outputs of two linear transformations through element-wise multiplication, which can be expressed as follows:

(1) $$O=\left(W_{1}^{T}X\right)⊙\left(W_{2}^{T}X\right)$$

To further illustrate the essence of the Star operation, after incorporating the bias term into the weights for simplification, the operation can be expanded as:

(2) $$w_{1}^{T}x*w_{2}^{T}x=\sum_{i=1}^{d}\sum_{j=1}^{d}\left(w_{1}^{i}w_{2}^{j}\right)\left(x^{i}x^{j}\right)$$

where (*O*) denotes the output feature of the Star module, and ($W_{1}^{T}X$) and ($W_{2}^{T}X$) represent two independent linear projections of the input feature. ($w_{1}^{i}w_{2}^{j}$) denote the (*i*)-th and (*j*)-th components of the weight vectors (*W*_1_) and (*W*_2_), respectively. Equation (2) can be expanded into a linear combination of (*d*^2^) cross terms, indicating that the Star operation implicitly represents the input in a (*d*^2^)-dimensional polynomial feature space.

It should be noted that the MNS module is not intended to introduce a completely new basic convolutional operator. Its multi-path aggregation structure is inspired by MANet, and the nonlinear interaction branch is derived from the Star Block. The task-specific design of MNS lies in embedding the Star-based nonlinear branch into a multi-path feature aggregation framework at the feature extraction stages where small pest structures are easily weakened. This design is motivated by the characteristics of insect pest monitoring lamp images, where pest targets are usually small, partially occluded, morphologically diverse, and visually similar across categories. Therefore, MNS aims to strengthen fine-grained texture interaction and multi-scale representation rather than simply replacing one generic block with another.

**Figure 5:**
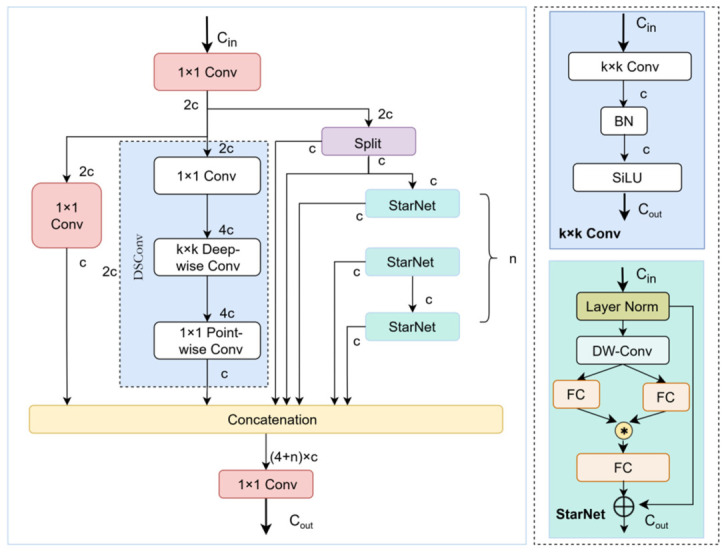
Structure of the MNS module. The symbol “*” denotes element-wise multiplication between the outputs of the two FC layers.

As shown in Figure 5, the MNS module first applies a (1 × 1) convolution to the input feature for initial transformation, followed by three parallel branches. The first branch employs standard convolution to extract basic semantic features. The second branch adopts depthwise separable convolution (DSConv) for lightweight spatial encoding. The third branch introduces a multi-stage cascaded Star module, in which each stage performs implicit high-order interactions through element-wise multiplication, while residual connections are used to stabilize training. The outputs of the three branches are concatenated along the channel dimension and then fused and compressed by a (1 × 1) convolution. Without changing the input and output dimensions of the original module, MNS improves the network’s recognition capability for small targets and challenging samples through an implicit high-dimensional feature construction mechanism.

#### 2.3.2. C2iBRA Module

The C2-based Position-Sensitive Attention (C2PSA) module in YOLO11 adopts a spatial attention mechanism with a fixed pattern. Its static spatial partitioning strategy is difficult to adapt to the dynamically varying spatial distribution of pests in monitoring images, and it also introduces additional computational overhead, which is unfavorable for deployment on edge devices. To address this issue, this study proposes a C2iBRA module for pest monitoring scenarios by incorporating the bi-level routing attention mechanism of BiFormer [24]. The proposed C2iBRA module is used to replace the original C2PSA module in the neck of YOLO11, thereby transforming the attention mechanism from static spatial partitioning to dynamic content-aware attention.

The BRA mechanism itself is adopted from BiFormer and serves as a general content-aware attention mechanism. In this study, the task-specific adaptation is reflected in its integration with a scale-aware preprocessing module before attention computation and its replacement of the original fixed-pattern attention unit in C2PSA. This modification is motivated by the pest monitoring lamp scenario, in which pest targets appear at different scales and are often disturbed by background stains, insect fragments, uneven illumination, and overlapping bodies. The scale-aware preprocessing enhances multi-scale spatial cues, while BRA dynamically selects more relevant regions for attention computation. Thus, C2iBRA is designed to improve foreground localization and background suppression in complex pest monitoring images.

**Figure 6:**
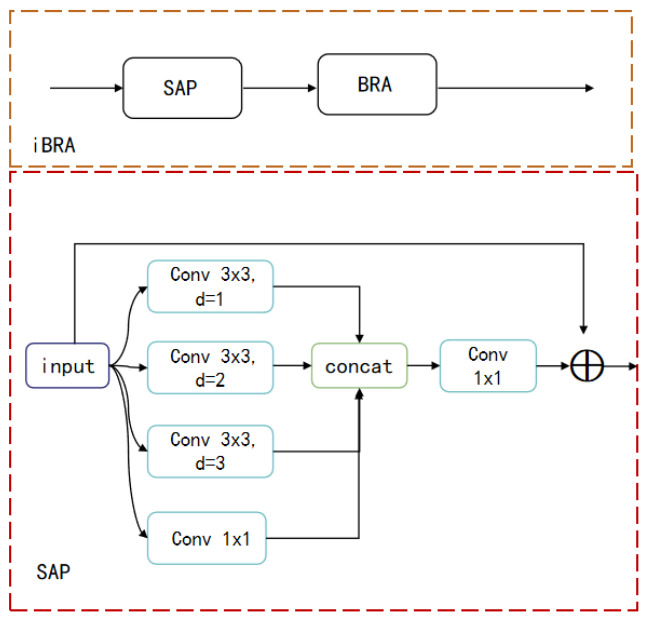
Structure of the iBRA module.

Considering the significant scale variations among pests in images captured by insect pest monitoring lamps, an iBRA module is designed as the core component of C2iBRA. As shown in Figure 6, iBRA consists of a Scale-Aware Preprocessing (SAP) module [25] followed by a BRA module. SAP adopts a multi-branch parallel dilated convolution structure, in which the input feature is fed into four parallel branches. Three branches employ (3 × 3) dilated convolutions with dilation rates of 1, 2, and 3, respectively, enabling progressive multi-scale feature extraction from local details to global contextual information. The fourth branch uses a (1 × 1) convolution to preserve the original spatial information. The outputs of all branches are concatenated along the channel dimension and then passed through a (1 × 1) projection convolution to perform cross-channel interaction and feature reorganization. A residual connection with the original input is further introduced to enrich scale diversity while effectively alleviating the vanishing gradient problem.

**Figure 7:**
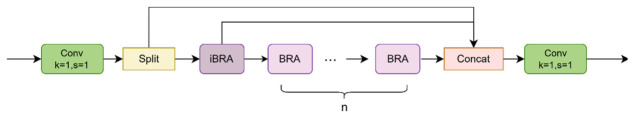
Structure of the C2iBRA module.

As shown in Figure 7, the overall structure of C2iBRA is similar to that of C2PSA, but the original attention unit is replaced with a more lightweight BRA attention module. BRA adopts a bi-level routing mechanism, which first selects the most relevant key–value pairs at the coarse-grained regional level and then performs attention computation at the fine-grained pixel level. In this way, computational complexity is significantly reduced while maintaining accurate perception. Specifically, C2iBRA first applies a (1 × 1) convolution for channel compression and then splits the feature into two branches along the channel dimension. One branch sequentially passes through the iBRA module and (N) cascaded BRA modules for deep semantic mining, while the other branch serves as a shortcut connection to maintain smooth gradient flow. The two branches are then concatenated along the channel dimension and fused by a (1 × 1) convolution to generate the output feature. This design enhances the model’s dynamic perception capability for multi-scale pest targets while effectively controlling the parameter size, making it more suitable for practical deployment in resource-constrained scenarios.

### 2.4. Semantic-Guided Adaptive Slicing Inference Strategy for High-Resolution Images

To further improve the applicability of YOLO11-MSC to original high-resolution images captured by insect pest monitoring lamps, this study designs a semantic-guided adaptive slicing inference strategy, termed SGAS. This strategy does not modify the model architecture. Instead, it transfers the detection capability learned from sliced images to full-image inference scenarios through a four-stage pipeline consisting of coarse detection, slicing, refined detection, and result fusion.

Specifically, in the first stage, YOLO11-MSC is employed as the coarse detector to perform detection on the original high-resolution image using a low confidence threshold, thereby retaining as many potential pest regions as possible. To prioritize the recall of small-scale and low-contrast pest targets, the confidence threshold in the coarse detection stage is set to 0.005. Subsequently, non-maximum suppression (NMS) is applied to the coarse detection results, with the IoU threshold set to 0.50, to remove highly overlapping candidate boxes and reduce redundant slicing. The centers of the candidate boxes retained after NMS are then used as slicing centers to crop local image patches of 960 × 960 pixels. This process maintains consistency between the object-centered prior used during training and the slices generated during inference, thereby reducing the redundant computation caused by processing large numbers of invalid background regions in fixed sliding-window slicing.

In the second stage, the semantically guided local slices are fed into the YOLO11-MSC-refined detector trained on the Insect35_OCAS dataset to obtain local detection results. Because the predicted bounding-box coordinates are defined in the local slice coordinate system, they are mapped back to the global coordinate system according to the offset of the upper-left corner of each slice in the original image. Since the same pest target may be covered by multiple overlapping slices and consequently detected more than once, Weighted Boxes Fusion (WBF) is applied after refined detection and global coordinate remapping to merge overlapping predictions from different slices, with the IoU threshold likewise set to 0.50. Finally, the pest categories, spatial locations, and quantity statistics for the entire image are obtained. This inference pipeline does not alter the model architecture and enables the detection capability learned from sliced data to be transferred to original high-resolution pest monitoring images, thereby providing a complete full-image detection solution for practical pest monitoring.

### 2.5. Model Training

#### 2.5.1. Training Platform and Experimental Settings

The experimental environment configuration is summarized in Table 1, while the network parameter settings are listed in Table 2. The dataset was divided into training, validation, and test sets at a ratio of 7:2:1. To accurately evaluate the effectiveness of the slicing strategy itself, only basic image augmentation methods were used during training.

#### 2.5.2. Evaluation Metrics

The evaluation metrics used in this study include precision, recall, average precision (AP), mean average precision (mAP) [26], the number of model parameters, and computational cost measured in giga floating-point operations (GFLOPs).

Precision represents the proportion of correctly predicted positive samples among all samples predicted as positive, reflecting the accuracy of the model in predicting a specific pest category. It is calculated as follows:

(3) $$Precision=\frac{TP}{TP+FP}$$

Recall represents the proportion of correctly detected positive samples among all actual positive samples, measuring the detection capability of the model for a given pest category. It is defined as:

(4) $$Recall=\frac{TP}{TP+FN}$$

where (*TP*) denotes the number of correctly detected positive samples, (*FP*) denotes the number of negative samples incorrectly classified as positive, and (*FN*) denotes the number of positive samples missed by the model.

Average precision (*AP*) measures the area under the Precision–Recall (P–R) curve for a single category and is used to evaluate the recognition quality of the model for that category. It can be expressed as:

(5) $$AP=\sum_{k=1}^{N}P\left(k\right)\Delta r\left(k\right)$$

Mean average precision (mAP) is calculated by averaging the *AP* values over all categories, thereby providing a comprehensive evaluation of the overall detection performance of the model. It is formulated as:

(6) $$mAP=\frac{1}{k}\sum_{i=1}^{k}AP_{i}$$

where (K) denotes the number of pest categories. In this study, (K = 35), corresponding to the 35 agricultural pest categories in the dataset.

The number of model parameters reflects the model size, while GFLOPs represent the total number of floating-point operations required during inference, which is used to measure the computational complexity and resource consumption of the model.

## 3. Results and Analysis

### 3.1. Detection Performance Comparison of Different Backbone Networks

To verify the effectiveness of the MNS module, YOLO11n was used as the baseline model in this study. The proposed MNS module was compared with several representative feature enhancement modules, including C3k2-MambaOut, C3k2-DynamicConv, C3k2-EMSCP, MAN, MAN-FasterCGLU, and MAN-GCConv. To ensure a fair comparison, all modules were embedded at the same positions and trained under identical experimental settings. Specifically, the third and fourth C3k2 modules in the backbone were replaced, and the corresponding C3k2 modules in the feature fusion stage were also substituted. The experimental results are presented in Table 3.

As shown in Table 3, the YOLO11n baseline model achieved an mAP, precision, and recall of 93.9%, 90.8%, and 91.8%, respectively. After introducing different modules, the detection performance of the model was improved to varying degrees. Among them, the C3k2-series modules increased the mAP by 0.2–0.4 percentage points, indicating relatively limited overall gains. In contrast, the MAN-series modules achieved better performance, suggesting that multi-path feature aggregation can enhance the model’s representation capability for multi-scale pest targets.

The proposed MNS module achieved the best overall results, with an mAP, Precision, and Recall of 95.0%, 91.9%, and 93.5%, respectively, representing improvements of 1.1, 1.1, and 1.7 percentage points over the baseline model. These results indicate that the MNS module realizes implicit high-order nonlinear interaction through element-wise multiplication, which strengthens the feature representation of fine-grained textures and small targets while effectively extracting spatial details and deep semantic information of pest objects.

In terms of model complexity, the number of parameters and the computational cost of MNS were 3.4 M and 7.9 GFLOPs, respectively. Its computational cost was lower than that of MAN and MAN-GCConv, both of which required 8.4 GFLOPs, while achieving higher mAP and recall. This demonstrates that the MNS module achieves a favorable balance between detection accuracy and computational overhead. Overall, MNS can effectively address the challenges of large target-scale variations, overlapping insect bodies, and strong background interference in insect pest monitoring lamp images while maintaining relatively low model complexity, indicating its potential for practical deployment.

### 3.2. Heatmap Comparison Analysis of Different Modules

**Figure 8:**
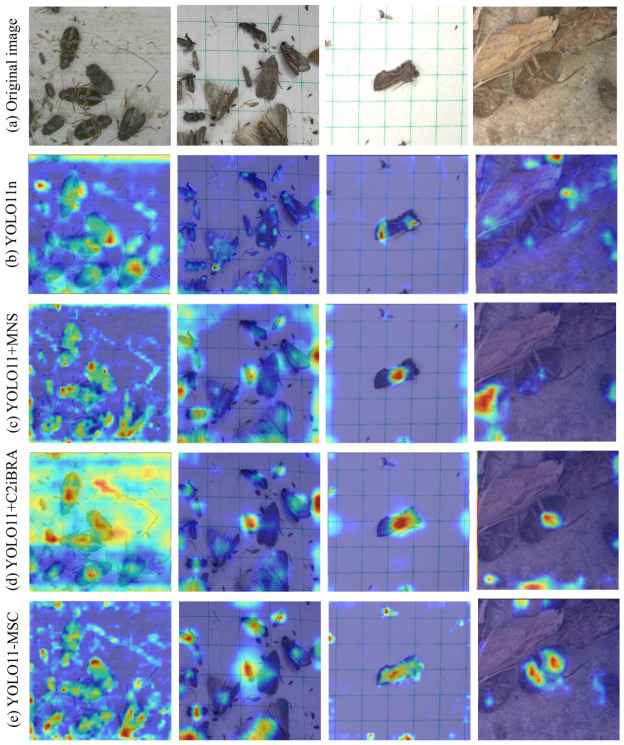
Comparison of Grad-CAM heatmap visualization results.

To evaluate the guidance effect of each improved module on the model’s feature-attention regions, Gradient-weighted Class Activation Mapping (Grad-CAM) was used to generate heatmaps, thereby visually presenting the attention distribution of the model on the input images. The results are shown in Figure 8, where the first to fourth columns correspond to borers, diamondback moth, cutworm, and rusty backswimmer, respectively. For the original YOLO11n model, the activation regions were relatively scattered, and part of the responses were mixed with background regions. In scenarios involving small-scale pests, such as the diamondback moth, and complex backgrounds, the distinction between target-related and background-related attention was insufficient, making it difficult for the model to effectively extract discriminative core features.

After introducing the MNS module, the activation responses of the model to key local features, such as pest heads and wing veins, were significantly enhanced, indicating an improved capability for capturing fine details. However, its ability to suppress background interference remained limited. With the addition of the C2iBRA module, the activation regions became more concentrated on the pest bodies, and the responses in background-irrelevant regions were effectively suppressed, resulting in clearer target contours under complex background conditions.

The YOLO11-MSC model exhibited the best heatmap visualization performance. The red high-activation regions accurately covered the pest bodies. For large-scale pests such as cutworms, medium-scale pests such as rusty backswimmers, and small-scale pests such as diamondback moths, the model could clearly highlight complete contours and key feature regions, while almost no invalid activation appeared in the background regions. The experimental results indicate that the MNS and C2iBRA modules are functionally complementary: the former strengthens multi-scale feature extraction, whereas the latter improves background suppression and attention focusing. Their synergistic effect enhances the model’s feature recognition capability in complex pest monitoring scenarios.

### 3.3. Ablation Analysis of the YOLO Model

To verify the effectiveness of each improved module, modular ablation experiments were conducted using YOLO11n as the baseline model. In the experiments, “√” indicates that the corresponding improved module was introduced, whereas “–” indicates that the module was not used. The results are presented in Table 4. The baseline model achieved an mAP, precision, and recall of 93.9%, 90.8%, and 91.8%, respectively, with 2.7 M parameters and 6.4 GFLOPs.

After introducing the C2iBRA module, the mAP increased to 94.6%, while Precision and Recall improved by 1.4 and 0.8 percentage points, respectively. Meanwhile, both the number of parameters and computational cost decreased slightly. This indicates that C2iBRA effectively improves the recognition capability for target features by enhancing attention focusing and background suppression without increasing the computational burden.

When the MNS module was introduced, the model performance improved more significantly. The mAP reached 95.0%, and precision and recall increased by 1.1 and 1.7 percentage points, respectively. The number of parameters and computational cost increased to 3.4 M and 7.9 GFLOPs, respectively. These results suggest that MNS effectively overcomes the limitations of the C3K2 module in terms of receptive field modeling and linear feature fusion by introducing multi-path heterogeneous feature aggregation and high-order nonlinear interaction, thereby enhancing the feature representation capability for multi-scale targets with complex morphologies.

When the two modules worked collaboratively, the model achieved the best performance, with an mAP, precision, and recall of 95.2%, 91.8%, and 93.0%, respectively, representing improvements of 1.3, 1.0, and 1.2 percentage points over the baseline model. The computational cost was reduced to 7.8 GFLOPs. These results demonstrate that YOLO11-MSC achieves high detection accuracy while maintaining a lightweight structure.

**Figure 9:**
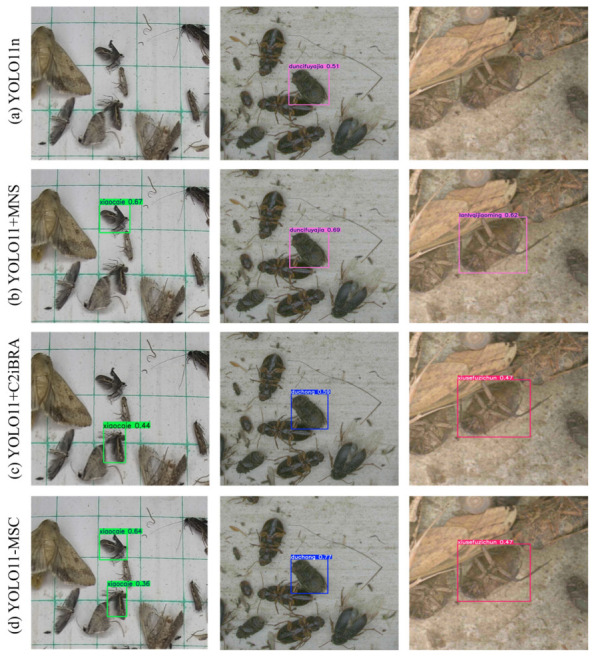
Visual comparison of test results.

To evaluate the practical detection performance of the improved modules, representative pest test samples with typical characteristics such as small object sizes and background interference were further selected for visual comparison. The results are shown in Figure 9, where the first to third columns correspond to the diamondback moth, borer beetle, and rusty backswimmer, respectively. In the baseline test, the original YOLO11n exhibited obvious missed detections and false detections because the key representations of small-scale pests were not sufficiently prominent, and the body colors of some pests were highly similar to stains on the background plate.

In the ablation comparison, YOLO11 + MNS enhanced the transmission of fine-grained features of small targets by constructing a multi-path feature aggregation network, increasing the detection confidence of diamondback moth to 67%. YOLO11 + C2iBRA improved the discriminative capability between targets and backgrounds through its attention focusing mechanism, effectively reducing missed detections of rusty backswimmer. Further analysis shows that YOLO11-MSC demonstrates strong robustness in both multi-scale and highly disturbed scenarios. It can effectively detect tiny pest bodies missed by the original model and achieves more accurate boundary regression for targets in complex backgrounds. The above visual evaluation, together with the quantitative metric analysis, further verifies the effectiveness of the proposed model in practical agricultural pest monitoring tasks.

### 3.4. Comparative Analysis of Detection Results for Different Models

To comprehensively evaluate the detection performance, lightweight advantage, and module generalizability of the proposed YOLO11-MSC model, several representative object detection models, including YOLOv8n, YOLOv10n, YOLO11n, YOLO13n, RT-DETR, and Faster R-CNN, were selected for comparative experiments on the Insect35_OCAS dataset. In addition, to further verify the applicability of the proposed MSC structure to different YOLO-based detectors, the MSC structure was transferred to YOLO13n to construct YOLO13-MSC. All comparison models were sorted in ascending order according to the number of parameters, allowing a more intuitive analysis of the trade-off among detection accuracy, model size, and computational cost. The experimental results are shown in Table 5.

As shown in Table 6, YOLOv10n has the smallest number of parameters, with only 2.3 M parameters and 6.7 GFLOPs. However, its mAP@0.5 is 93.2%, which is still lower than those of YOLO11n and YOLO13n. YOLO13n has 2.5 M parameters and 6.2 GFLOPs, and achieves an mAP@0.5 of 94.1%, showing relatively good performance among the original lightweight YOLO models. YOLOv8n obtains an mAP@0.5 of 93.6%, which is slightly lower than those of YOLO11n and YOLO13n, indicating that different lightweight YOLO models still exhibit certain performance differences in complex small pest detection scenarios.

Compared with the original YOLO11n, the proposed YOLO11-MSC improves the mAP@0.5 from 93.9% to 95.2%, corresponding to an increase of 1.3 percentage points, while achieving a Precision of 91.8% and a Recall of 93.0%. In terms of model complexity, YOLO11-MSC contains only 3.4 M parameters and 7.8 GFLOPs, maintaining a relatively small model size and low computational cost. These results indicate that the proposed improved structure can effectively enhance pest detection accuracy without significantly increasing model complexity.

Furthermore, after transferring the MSC structure to YOLO13n, YOLO13-MSC achieves an mAP@0.5 of 95.5%, which is 1.4 percentage points higher than that of YOLO13n, and obtains the highest Recall of 93.4%. This result demonstrates that the proposed MSC structure is not only applicable to YOLO11n but can also be transferred to other YOLO-based detectors, showing good generalizability. In comparison, RT-DETR achieves a Precision of 92.5%, indicating a strong ability to suppress false positives. However, its parameter count and computational cost reach 32.0 M and 103.6 GFLOPs, respectively. Faster R-CNN further increases the parameter count and computational cost to 41.4 M and 134.6 GFLOPs, respectively, but achieves only 91.8% mAP@0.5. This indicates that the traditional two-stage detector does not show an accuracy advantage in the small pest detection task using insect pest monitoring lamp images and has a relatively high deployment cost.

Overall, YOLO13-MSC achieves the highest detection accuracy, validating the effectiveness and generalizability of the proposed MSC structure. Meanwhile, YOLO11-MSC maintains near-optimal detection performance with fewer parameters and a lower computational cost, achieving a better balance between detection accuracy and lightweight deployment. Therefore, YOLO11-MSC is more suitable for insect pest detection tasks using monitoring lamp images under resource-constrained conditions.

**Figure 10:**
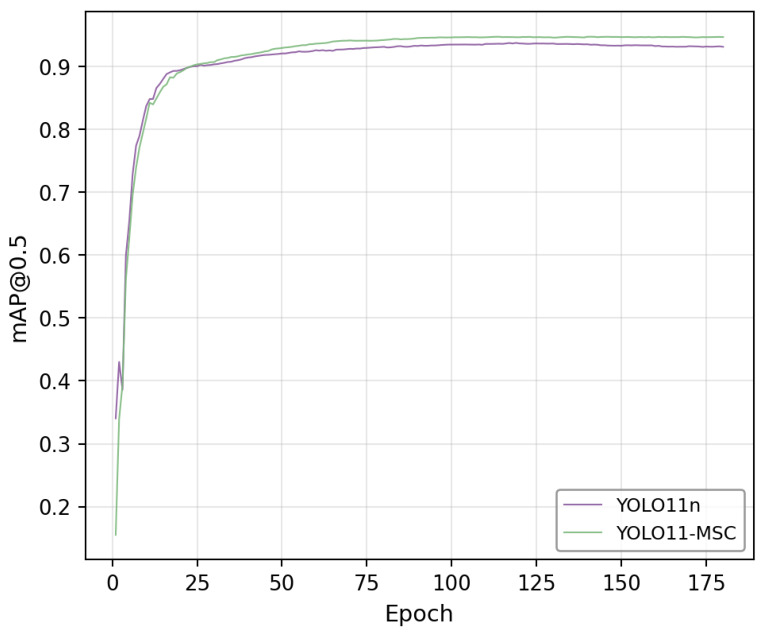
mAP@0.5 curves of YOLO11n and YOLO11-MSC.

**Figure 11:**
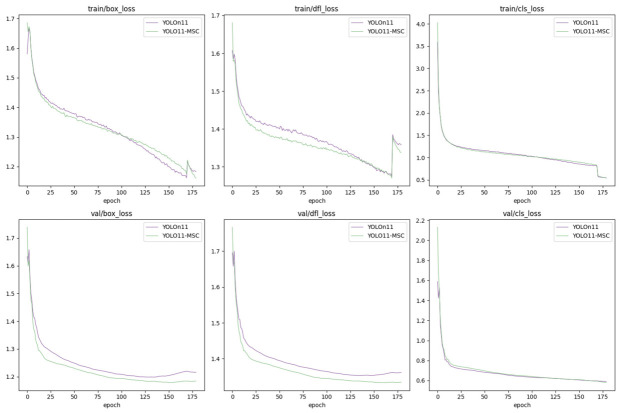
Training and validation loss curves of YOLO11n and YOLO11-MSC.

To further analyze the dynamic convergence behavior of the improved algorithm during training, the mAP@0.5 curves of YOLO11-MSC and the baseline YOLO11n over 180 epochs were plotted, as shown in Figure 10. In addition, the training and validation loss curves are presented in Figure 11. As shown in the figures, during the early training stage from 0 to 25 epochs, the localization loss (box_loss), distribution focal loss (dfl_loss), and classification loss (cls_loss) of both YOLO11-MSC and the baseline YOLO11n decreased rapidly, while the corresponding mAP@0.5 curves increased sharply. This indicates that both models quickly learned effective feature representations in the initial training stage.

As training progressed into the middle stage from 25 to 165 epochs, the slopes of the curves gradually became smoother, indicating that the models entered a relatively stable parameter refinement and high-level feature learning stage. During this period, YOLO11-MSC consistently achieved higher mAP@0.5 values than the baseline model. In terms of validation loss, the loss trajectories of YOLO11-MSC were generally lower and smoother than those of YOLO11n. This advantage was particularly evident in val/box_loss and val/dfl_loss, which are closely related to bounding box regression accuracy. Moreover, no obvious upward trend caused by overfitting was observed in the late training stage.

These results suggest that the heterogeneous multi-path aggregation of the MNS module and the attention perception mechanism of C2iBRA help the model suppress background noise in complex agricultural scenarios and extract more robust geometric and structural representations of pest targets. In addition, around the 170th epoch, the training strategy triggered the Close Mosaic mechanism, resulting in a short-term fluctuation in the training loss. After this adjustment, the model further adapted to the original image distribution, and its classification and localization performance continued to improve before converging at the end of training. Overall, YOLO11-MSC demonstrates stronger generalization ability, higher optimization efficiency, and better convergence stability than the baseline YOLO11n.

### 3.5. High-Resolution Full-Image Inference Analysis

To comprehensively evaluate the effectiveness of the proposed SGAS inference strategy in high-resolution insect pest monitoring images, both quantitative metrics and qualitative visualizations are analyzed in this study.

As shown in Table 6, comparative experiments were conducted on the original Insect35 dataset using YOLO11n, YOLO11-MSC, and YOLO11-MSC + SGAS. The results indicate that YOLO11n achieves a mAP, precision, and recall of 56.7%, 58.6%, and 58.2%, respectively, when directly applied to full-resolution images, demonstrating limited detection performance. This is mainly attributed to the high resolution of insect trap images, where small pest objects occupy only a very small proportion of the entire image. Direct resizing leads to severe loss of fine-grained texture and structural information, resulting in missed and false detections.

After introducing the MNS and C2iBRA modules, YOLO11-MSC significantly improves performance, achieving a mAP of 84.3%, a precision of 80.1%, and a recall of 86.2%. This indicates that the proposed architectural enhancements effectively strengthen the feature representation capability for small-scale pest objects in high-resolution images. Furthermore, by incorporating the SGAS inference strategy, YOLO11-MSC + SGAS further improves performance, reaching a mAP of 86.4%, a Precision of 81.2%, and a Recall of 90.1%, which corresponds to gains of 2.1, 1.1, and 3.9 percentage points over YOLO11-MSC, respectively.

**Figure 12:**
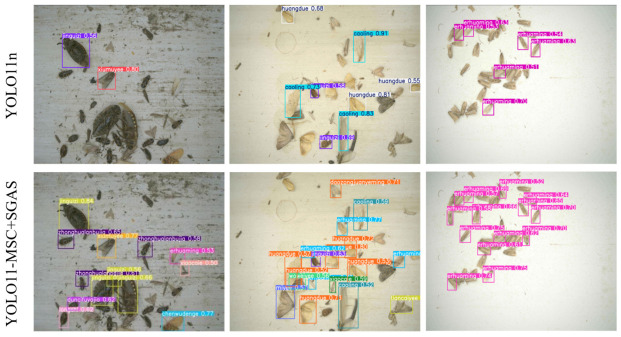
Comparison of detection results on high-resolution insect pest monitoring images.

To further provide an intuitive comparison of detection performance in high-resolution scenarios, representative insect trap images are visualized in Figure 12. As shown in the figure, YOLO11n exhibits severe missed detections, particularly for small-scale pests and regions with background patterns similar to target appearances, resulting in sparse and inaccurate bounding boxes. In contrast, YOLO11-MSC, enhanced by the MNS module and the C2iBRA module, demonstrates significantly improved sensitivity to small objects and is able to detect most major pest instances while effectively reducing background-induced false positives. However, it still suffers from partial omissions in densely populated regions.

In comparison, YOLO11-MSC + SGAS achieves the best overall performance. It not only accurately detects small-scale pests in densely distributed regions but also significantly improves recall and detection completeness while reducing false detections under complex backgrounds. These results demonstrate that the semantic-guided slicing strategy effectively suppresses irrelevant background interference and enables more sufficient feature extraction of small objects within local regions, thereby improving the recall ability for both small and densely distributed targets.

Although the slicing-based inference increases the per-image inference time from 0.014 s to 0.061 s, such overhead is acceptable in practical insect monitoring scenarios, as insect trap systems typically operate at relatively low acquisition frequencies and do not require strict real-time response at the second level.

Overall, the results demonstrate that SGAS effectively bridges the gap between slice-based training and full-image inference, significantly improving the stability and applicability of YOLO11-MSC in high-resolution insect pest detection tasks.

**Table 6:** Performance comparison with the SGAS inference strategy.

Model	mAP (%)	Precision (%)	Recall (%)	Params (M)	Detection Time (s)	GFLOPs
YOLO11n	56.7	58.6	58.2	2.5	0.002	6.4
YOLO11-MSC	84.3	80.1	86.2	3.4	0.014	7.8
YOLO11-MSC + SGAS	86.4	81.2	90.1	3.4	0.061	7.8

## 4. Discussion

The experimental results indicate that YOLO11-MSC achieves a favorable balance between detection accuracy and lightweight deployment for small pest detection in insect pest monitoring lamp images. On the Insect35_OCAS dataset, YOLO13-MSC achieved the highest detection accuracy, which further confirms the generalizability and effectiveness of the proposed MSC module when transferred to different YOLO-based detectors. However, compared with YOLO13-MSC, YOLO11-MSC requires fewer parameters and a lower computational cost while maintaining competitive detection performance. Therefore, YOLO11-MSC is considered more suitable for lightweight deployment scenarios where both detection accuracy and computational efficiency are important.

This result is closely related to the characteristics of insect monitoring lamp images. In such images, pest targets usually occupy only a small area, and many insects appear with similar colors, incomplete bodies, overlapping regions, or complex background stains. These factors make it difficult for general detectors to extract stable and discriminative features. Similar difficulties have also been reported in previous studies on light-trap pest detection, especially for dense targets, scale variation, and visually similar categories.

The improvement of YOLO11-MSC is mainly derived from the combined design of OCAS, MNS, and C2iBRA. OCAS crops image patches around pest targets, which increases the relative size of small insects in the input image and reduces useless background information. This is helpful because directly resizing high-resolution monitoring images may weaken the texture and boundary details of small pests. The MNS module strengthens feature extraction through multi-path aggregation and nonlinear interaction, making the model more sensitive to fine-grained pest structures. In contrast, C2iBRA mainly improves the attention distribution of the network, allowing the model to focus more on pest bodies while suppressing background responses. The Grad-CAM results also support this observation, as YOLO11-MSC shows more concentrated activation regions than the baseline model.

The SGAS inference strategy further improves the use of YOLO11-MSC on original high-resolution images. Instead of using fixed sliding windows, SGAS first locates potential pest regions and then performs local slicing and refined detection. This process reduces unnecessary background regions and makes full-image inference more consistent with slice-based training. It should be noted that the 95.2% mAP@0.5 achieved by YOLO11-MSC on the Insect35_OCAS sliced test set and the 86.4% mAP@0.5 obtained on the original full-image dataset using the SGAS inference strategy do not correspond to identical evaluation scenarios. The former was evaluated on object-centered sliced images, where pest targets are generally located near the center of the image and occupy a relatively larger proportion of the input. In contrast, the latter was evaluated on original high-resolution insect pest monitoring lamp images, where pest targets are smaller and background interference is more complex, thereby substantially increasing the detection difficulty.

The performance drop can be attributed to several factors. First, pest targets occupy a much smaller proportion of the original full images, which makes their texture, edge, and morphological features more likely to be weakened. Second, the original insect pest monitoring lamp images contain considerable background interference, including stains on the lamp board, insect fragments, target adhesion, occlusion, and uneven illumination. These factors increase the difficulty for the model to distinguish pest bodies from background regions. Third, the SGAS inference process involves multiple steps, including candidate region localization, local slicing, coordinate mapping, and prediction fusion. Errors introduced in any of these steps may affect the final full-image detection performance. In addition, some extremely small, incomplete, or boundary-located pest targets may not be fully covered by the generated candidate regions, resulting in missed detections.

Therefore, the 86.4% mAP@0.5 obtained on the original full-image dataset better reflects the detection difficulty in practical insect pest monitoring lamp scenarios. Although this result is lower than that obtained on the sliced test set, SGAS still avoids the loss of small-object information caused by directly resizing the entire high-resolution image and enables the model to perform inference on original full images. Future work will further optimize candidate region generation, slice boundary handling, and prediction fusion strategies to reduce the performance gap between sliced-image evaluation and original full-image inference.

From the perspective of architectural novelty, the proposed modules should be understood as task-oriented adaptations of existing components rather than entirely new basic operators. The multi-path structure in MNS is related to MANet; the nonlinear interaction branch is derived from the Star Block, and the BRA mechanism in C2iBRA is adopted from BiFormer. However, the motivation of these integrations is closely related to the practical pain points of insect pest monitoring lamp images, including small target scales, fine-grained inter-class similarity, incomplete insect bodies, dense distributions, and strong background interference. The experimental results and visual analyses show that MNS mainly improves fine-grained and multi-scale feature representation, whereas C2iBRA mainly contributes to target-focused attention and background suppression. Therefore, the contribution of this work lies in the scenario-driven adaptation, integration, and validation of these components for high-resolution pest monitoring images, together with the OCAS and SGAS strategies for slice-based training and full-image inference.

This study still has several limitations. First, although the dataset contains images collected from different regions and seasons, it may not fully cover all practical field conditions, such as extreme illumination, differences among monitoring devices, severe insect adhesion, or rare pest categories. Therefore, future work should collect more field data to improve the robustness of the model. Second, although YOLO11-MSC has a small parameter count and a low computational cost, its lightweight property in the current study was mainly evaluated from the perspective of model complexity. Practical deployment experiments on edge devices, such as Jetson-series platforms, have not yet been conducted. Therefore, future work will further evaluate the inference speed, memory consumption, energy efficiency, and detection stability of YOLO11-MSC on edge devices under real insect pest monitoring lamp scenarios.

## 5. Conclusions

In this study, the proposed YOLO11-MSC model demonstrated favorable overall performance in pest detection tasks for agricultural light-trap monitoring systems. Specifically, the introduced MNS module effectively enhanced the model’s feature extraction capability for multi-scale, small-scale, and morphologically complex pest targets. The constructed C2iBRA module further improved the model’s attention to target core regions and suppressed redundant interference in complex backgrounds. Based on systematic comparative experiments, ablation experiments, and visual analyses, the following conclusions can be drawn.

(1) Compared with improved modules such as C3k2-MambaOut, C3k2-DynamicConv, C3k2-EMSCP, MAN, MAN-FasterCGLU, and MAN-GCConv, the proposed MNS module achieved the best overall performance in the pest detection task. The MNS-based model achieved an mAP, precision, and recall of 95.0%, 91.9%, and 93.5%, respectively, representing improvements of 1.1, 1.1, and 1.7 percentage points over the YOLO11n baseline model.
(2) The ablation experimental results showed that the YOLO11-MSC model achieved an mAP, precision, and recall of 95.2%, 91.8%, and 93.0%, respectively. These results verify the effectiveness and complementarity of the MNS and C2iBRA modules in enhancing feature representation and attention focusing.
(3) In comparison with YOLO11n, YOLO13n, and RT-DETR, YOLO11-MSC improved the mAP by 1.3, 1.1, and 0.5 percentage points, respectively. Meanwhile, the number of parameters and computational cost of YOLO11-MSC were only 3.4 M and 7.8 GFLOPs, respectively, which were significantly lower than those of RT-DETR, which contained 32 M parameters and required 103.6 GFLOPs. Furthermore, after combining YOLO11-MSC with the SGAS inference strategy, the mAP, precision, and recall on the original high-resolution full-image dataset reached 86.4%, 81.2%, and 90.1%, respectively. This indicates that SGAS can effectively bridge slice-based training and full-image inference, thereby improving the detection stability and practical applicability of the model in real insect pest monitoring lamp images.

Overall, YOLO11-MSC can effectively reduce missed detections of small targets, inter-class false detections, and background interference, demonstrating good detection stability and practical deployment value in complex insect pest monitoring lamp images. The results of this study provide effective technical support for intelligent agricultural pest monitoring and precision pest control.

## Figures and Tables

**Table 1:** Experimental environment configuration.

Category	Version
Operating System	Ubuntu 22.04
GPU	NVIDIA Quadro RTX 5000
CPU	Intel(R) Core(TM) i9-10900K
RAM	125GB
Python	3.8
Pytorch	2.3.0
CUDA	12.1
Cudnn	8.9.2

**Table 2:** Network Parameter Settings.

Category	Parameter
Input_shape	640 × 640
Epochs	180
Batch_size	16
Initial learning rate	0.01
Optimizer	SGD

**Table 3:** Multi-index performance evaluation of different models.

Model	mAP (%)	Precision (%)	Recall (%)	Params (M)	GFLOPs
Baseline (YOLO11n)	93.9	90.8	91.8	2.7	6.4
C3k2-MambaOut	94.1	91.3	92.3	2.3	6.1
C3k2-DynamicConv	94.1	90.4	92.5	3.5	6.2
C3k2-EMSCP	94.3	91.5	92.8	2.5	6.3
MAN	94.8	91.2	93.2	3.7	8.4
MAN-FasterCGLU	94.7	91.8	92.2	3.1	7.2
MAN-GCConv	94.3	91.3	91.8	3.7	8.4
MNS (Ours)	95	91.9	93.5	3.4	7.9

**Table 4:** Ablation experiments on the improved modules.

Model	MNS	C2iBRA	mAP (%)	Precision (%)	Recall (%)	Params (M)	GFLOPs
YOLO11n	-	-	93.9	90.8	91.8	2.7	6.4
YOLO11n	-	√	94.6	92.2	92.6	2.6	6.3
YOLO11n	√	-	95	91.9	93.5	3.4	7.9
YOLO11n	√	√	95.2	91.8	93	3.4	7.8

**Table 5:** Comparison of detection performance of existing models.

Model	mAP (%)	Precision (%)	Recall (%)	Params (M)	GFLOPs
YOLOv10	93.2	90.6	91.4	2.3	6.7
YOLO13n	94.1	91	93	2.5	6.2
YOLO11n	93.9	90.8	91.8	2.7	6.4
YOLOv8	93.6	90.5	91.4	3.2	8.7
YOLO11-MSC	95.2	91.8	93	3.4	7.8
YOLO13-MSC	95.5	92.3	93.4	3.8	8.5
RTDETR	94.7	92.5	92.3	32	103.6
Faster R-CNN	91.8	89.5	89.1	41.4	134.6

## Data Availability

The data presented in this study are not publicly available because the dataset is still being used for ongoing research by members of our laboratory, and some raw monitoring images were collected through long-term field-deployed pest monitoring devices provided by a collaborating company. The data are available from the corresponding author upon reasonable request.

## Abbreviations

The following abbreviations are used in this manuscript:

MNS	Multi-path Nonlinear Star aggregation
OCAS	Object-Centric Adaptive Slicing
SGAS	Semantic-Guided Adaptive Slicing

## Appendix A

**Table A1:** Sample distribution of the Insect35_OCAS dataset.

Pest Number	Name of Pest	Chinese Label of Pest	Number of Images
1	Mole cricket	lougu	2596
2	Green lacewing	caoling	226
3	Yellow tussock moth	huangdue	361
4	Elegant lichen moth	youmeitaie	200
5	Rice green bug	daoluchun	341
6	Blunt-spined beetle	duncifuyajia	7028
7	Rotten-wood moth	xiumuyee	3409
8	Noctuid moth	woweiyee	1037
9	Diamondback moth	xiaocaie	795
10	Silver-striped moth	yinwenyee	551
11	Diving beetle	longshi	2332
12	Rice stem borer	erhuaming	2642
13	Cricket	xishuai	4694
14	Red bug	hongchun	9145
15	Meadow moth	maimuyeming	1042
16	Scarab beetle	jinguizi	16,612
17	Cutworm	dilaohu	3829
18	Rusty backswimmer	xiusefuzichun	270
19	White tiger moth	xingbaixuedenge	586
20	Tobacco cutworm	xiewenyee	437
21	Borer beetle	duchong	120
22	Armyworm	nianchong	292
23	Rice leaf folder	daozongjuanyeming	268
24	Noctuid moth	miyee	445
25	Beet armyworm	tiancaiyee	983
26	Olive-green moth	lanluqijiaoming	505
27	Yellow peach moth	taozhuming	692
28	Lichen moth	tutaie	851
29	Yellow-legged assassin bug	huangzuliechun	314
30	Black-and-white lichen moth	baiheihuataie	412
31	Burying beetle	zangjia	1345
32	White-striped moth	baitiaoyee	172
33	Dusty tiger moth	chenwudenge	1642
34	Cotton bollworm	mianlingchon	1508
35	Chinese ground beetle	zhonghualanbujia	1943
total	/	/	69,625
